# Proliferative Regeneration of Zebrafish Lateral Line Hair Cells after Different Ototoxic Insults

**DOI:** 10.1371/journal.pone.0047257

**Published:** 2012-10-18

**Authors:** Scott M. Mackenzie, David W. Raible

**Affiliations:** 1 Department of Biological Structure, University of Washington, Seattle, Washington, United States of America; 2 Graduate Program in Neurobiology and Behavior, University of Washington, Seattle, Washington, United States of America; Pomona College, United States of America

## Abstract

Sensory hair cells in the zebrafish lateral line regenerate rapidly and completely after damage. Previous studies have used a variety of ototoxins to kill lateral line hair cells to study different phenomena including mechanisms of hair cell death and regeneration. We sought to directly compare these ototoxins to determine if they differentially affected the rate and amount of hair cell replacement. In addition, previous studies have found evidence of proliferative hair cell regeneration in zebrafish, but both proliferation and non-mitotic direct transdifferentiation have been observed during hair cell regeneration in the sensory epithelia of birds and amphibians. We sought to test whether a similar combination of regenerative mechanisms exist in the fish. We analyzed the time course of regeneration after treatment with different ototoxic compounds and also labeled dividing hair cell progenitors. Certain treatments, including cisplatin and higher concentrations of dissolved copper, significantly delayed regeneration by one or more days. However, cisplatin did not block all regeneration as observed previously in the chick basilar papilla. The particular ototoxin did not appear to affect the mechanism of regeneration, as we observed evidence of recent proliferation in the majority of new hair cells in all cases. Inhibiting proliferation with flubendazole blocked the production of new hair cells and prevented the accumulation of additional precursors, indicating that proliferation has a dominant role during regeneration of lateral line hair cells.

## Introduction

Damage to auditory hair cells is associated with many factors, including exposure to various drugs, loud noise, and age-related degradation. Unfortunately, the majority of sensory hair cells do not regenerate in adult mammals [1] despite the potential of limited regeneration in the vestibular sensory epithelium [2]–[5] and in the auditory sensory epithelium of early post-natal animals [6], [7]. Progressive hearing loss can significantly impair daily routines and social interaction [8]. By contrast, regeneration of sensory hair cells has been observed in other non-mammalian vertebrates, including birds [9], 10, amphibians [11], [12], and fish [13]–[17].

Non-sensory supporting cells in the auditory and vestibular organs are the source of hair cell precursors, and two mechanisms of regeneration have been observed after hair cell damage. New hair cells are frequently derived from dividing precursors during the process of proliferative regeneration [18], [19]. Alternatively, new hair cells can also be generated by direct phenotypic conversion of supporting cells into hair cells without cell division, a process called transdifferentiation [20]–[26]. Determining how regeneration is initiated and regulated in these organisms may lead to treatments that restore lost hearing in humans.

The zebrafish lateral line system provides an excellent model to study the process of hair cell regeneration. Zebrafish larvae are mostly transparent, and the lateral line is easily accessed on the body surface. Lateral line hair cells exhibit morphology and function similar to inner ear hair cells [27], [28]. Moreover, they are susceptible to compounds toxic to inner ear hair cells (ototoxins) such as the aminoglycoside antibiotics neomycin and gentamicin [15], [29]–[33] as well as platinum derivatives used in chemotherapy such as cisplatin [32], [34], [35]. Lateral line hair cells are also susceptible to damage by water-borne copper [17], [36]. Zebrafish regenerate lateral line hair cells rapidly after damage, and almost all hair cells are replaced after 72 hours [15], [31], [37]–[39].

There are several lines of evidence that the type and extent of damage alter the course of hair cell regeneration. Differential exposure to copper affects the capacity for regeneration: treatment with low concentrations of copper elicits regeneration, while treatment with high concentrations prevents most hair cell replacement [17]. Exposure at moderate to high concentrations also damages support cells [40], preventing their differentiation into new hair cells. Cisplatin has been shown to have analogous effects on the regeneration of avian cochlear and vestibular sensory epithelia, killing hair cells and preventing both proliferative regeneration and interfering with direct transdifferentiation [41]. It has also been suggested that the extent of exposure affects the type of regeneration that occurs in the zebrafish lateral line system, with exposure to a low concentration of copper resulting in non-proliferative regeneration and treatment with a high concentration resulting in proliferative regeneration [38]. By contrast, most evidence suggests that proliferation is the dominant regenerative mechanism in the zebrafish lateral line following treatment with neomycin [37], [39], [42].

Taken together these data suggested that different types or levels of damage could elicit different responses with potentially distinct underlying molecular regulation. Here we revisit possible mechanisms of hair cell regeneration in the zebrafish lateral line system after exposure to several ototoxins. We compare the regenerative responses to copper, cisplatin, and the aminoglycosides neomycin and gentamicin, which we have previously suggested kill zebrafish lateral line hair cells by both distinct and overlapping mechanisms [33], [43]. We find under all conditions that hair cells regenerate predominantly using proliferative mechanisms irrespective of the type of toxin exposure.

## Materials and Methods

### Zebrafish Maintenance

Zebrafish (*Danio rerio*) embryos were obtained from pairings of *AB wild type or transgenic *Et(krt4:EGFP)sqet4* adult fish (“ET4:GFP fish;” ZDB-GENO-070702-7 [44], [45]; gift of V. Korzh) and maintained at 28.5°C in embryo medium (EM; 0.04 mM Na_2_HPO_4_, 0.15 mM KH_2_PO_4_, 1 mM MgSO_4_, 1 mM CaCl_2_, 0.5 mM KCl, 15 mM NaCl, 0.7 mM NaHCO_3_, pH 7.2). Larvae were fed live rotifers daily beginning at 4 days post-fertilization (dpf) with the exception of days when they were treated with an ototoxin. Experiments were conducted beginning at 3 or 5 dpf and were completed by 9 dpf. All animal procedures were approved by the University of Washington Institutional Animal Care and Use Committee, AWA number A3464-01.

### Treatment with Ototoxic Compounds

Embryos at 3 or 5 dpf were incubated for between 30 min and 10 hours in EM (control treatment), neomycin (100–200 μM; Sigma), copper(II) sulfate (0.3–30 μM; Fluka), gentamicin (50–200 μM; Sigma), or cisplatin (50 μM; Sigma). All solutions were prepared by serial dilution when appropriate. Larvae were rinsed twice in fresh EM after treatment then returned to fresh EM or another solution as described.

### Regeneration Assays

Regeneration was measured in two ways. Initial experiments used a semi-quantitative scoring technique described previously [15] using the vital dye FM 1-43FX (300 nM; Invitrogen), which selectively labels hair cells with functional mechanotransduction channels [30]. Larvae were incubated in FM 1-43FX for 10 min at various hours post-treatment (hpt, measured from the beginning of treatment), anesthetized in ethyl-*m*-aminobenzoate methanesulfonate (MESAB), and viewed with an epifluorescent dissecting microscope. Ten neuromasts of the anterior lateral line (IO1-4, M2, MI1, MI2, O2, SO1, and SO2) were scored using a semiquantitative scale where 0 = no signal, 1 = partial signal, and 2 = full signal for a total possible score of 20. Larvae were screened only once. In other experiments, individual hair cells from 7 neuromasts (IO4, M2, OP1, MI1, MI2, O1, and O2) were labeled with antibodies against parvalbumin as described below and counted using a Zeiss Axioplan 2 epifluorescent microscope. Eight larvae were assessed in each treatment group.

### Proliferation Assay

To detect evidence of recent cell proliferation, ET4:GFP larvae were incubated in fresh EM containing 5 mM 5-bromo-2′-deoxyuridine (BrdU), a thymine analog, for 23 h immediately after a 1 hour (h) treatment with an ototoxin. BrdU-containing medium was replaced with fresh EM at 24 hpt and maintained for an additional 48 h. One reason for labeling DNA synthesis only during the initial stage of regeneration is that BrdU is a toxic mutagen and might affect hair cell survival [46]. Larvae were collected at 72 hpt, fixed by immersion in 4% paraformaldehyde, and labeled with antibodies against parvalbumin and BrdU as described below. Hair cell counts assessed the incorporation of BrdU in 3 neuromasts per fish.

### Inhibition of Proliferation

To determine whether hair cell regeneration requires proliferation, larvae were incubated in fresh EM containing 5 μM flubendazole, which blocks microtubule assembly [47], until 24 or 48 hpt. Larvae were collected and labeled with antibodies against parvalbumin, GFP, or phosphohistone H3 (PHH3) as described below. Hair cell counts assessed 3 or 7 neuromasts per fish and 8 fish per group.

### Whole Mount Immunohistochemistry

Larvae were fixed in 4% paraformaldehyde and then rinsed in PBST (phosphate-buffered saline with 0.1% Tween-20) and ddH_2_O. To label mature hair cells, larvae were blocked in 5% goat serum and incubated in mouse anti-parvalbumin primary antibody (1∶500; Millipore) followed by goat anti-mouse Alexa Fluor 488 or 568 secondary antibody (1∶1,000; Invitrogen). To label GFP in ET4:GFP larvae, mouse (1∶1,000; UC Davis/NIH NeuroMab Facility, clone N86/38) or rabbit (1∶1,000; Invitrogen) anti-GFP primary antibody and goat anti-mouse or anti-rabbit Alexa Fluor 488 secondary antibody (1∶1,000) were used. To label PHH3, rabbit anti-PHH3 primary antibody (1∶500; Millipore) and goat anti-rabbit Alexa Fluor 568 secondary antibody (1∶1,000) were used. For the proliferation assay measuring incorporation of BrdU, larvae were subsequently rinsed in PBSDT (PBST with 1% DMSO) and 1 M HCl before being blocked in 10% goat serum again. Larvae were incubated in rat anti-BrdU primary antibody (1∶100; Abcam) followed by goat anti-rat Alexa Fluor 568 secondary antibody (1∶400). Larvae were viewed on a Zeiss LSM 5 confocal microscope using a 40X oil immersion objective. PASCAL image acquisition software was used to collect *z*-stacks of 3 neuromasts (MI1, O1, and O2) per fish in 1 μm sections, which were later analyzed using ImageJ.

### Figure Preparation and Statistics

Figures were prepared using GraphPad Prism 5 and Adobe Illustrator CS3. Images were edited using Adobe Photoshop CS3. In all figures, error bars represent standard deviation. Differences between groups and time points were analyzed using Prism 5 with a one- or two-way ANOVA followed by a Tukey or Bonferroni posttest.

## Results

### Robust Proliferative Regeneration Occurs after Treatment with Neomycin or Copper

**Figure 1:**
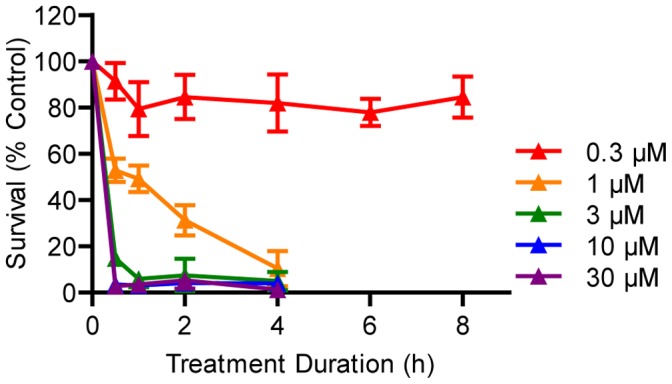
Rapid hair cell loss after treatment with water-borne copper. Wild type larvae were treated at 5 dpf with serial dilutions of copper(II) sulfate for a period of 30 min to 8 h. Hair cell death was rapid in most cases, although 0.3 μM copper had a minimal effect. *N* = 8 fish per group. Error bars are +/− SD.

We have previously shown that media composition alters hair cell toxicity [48]. To ensure copper treatments effectively killed hair cells under conditions comparable to those used with aminoglycoside antibiotics, we performed a dose response assay using 5 dpf wild type *AB larvae treated with concentrations of dissolved copper(II) sulfate ranging from 0.3–30 μM and continuous exposure times ranging from 30 min to 10 h. These data show a consistent relationship between concentration, duration of exposure, and hair cell death (Figure 1) that is similar to previous reports [17], [36]. While 1 μM copper killed nearly all hair cells within 4 h, higher concentrations were effective within 30–60 min. The interplay between concentration and time of treatment suggests that copper may act in a cumulative fashion to damage hair cells.

**Figure 2:**
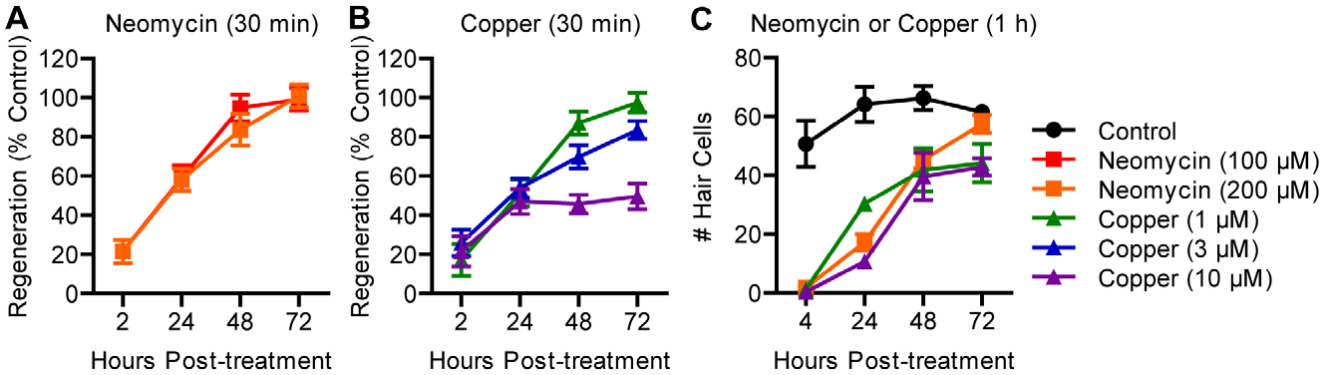
Copper may impair hair cell regeneration. (A, B) Wild type larvae were treated at 5 dpf with serial dilutions of neomycin or copper for 30 min. Hair cell regeneration was scored using FM 1-43FX and normalized to controls. Regeneration was incomplete in groups treated with 3 or 10 μM copper, but other groups recovered fully. *N* = 7 fish per group. (C) Individual counts of hair cells labeled with antibodies against parvalbumin confirmed incomplete regeneration in larvae treated for 1 h with copper. *N* = 8 fish per group. Error bars are +/− SD.

We next compared hair cell regeneration after treatment with copper or neomycin on larvae at 5 dpf, when neuromast development is largely complete. As reported previously [15], [39], we observed full regeneration after treatment with varying concentrations of neomycin (Figure 2A,C). In contrast, not all larvae treated with copper experienced full regeneration. Larvae treated with 1 μM copper for 30 min fully recovered, but larvae treated with 10 μM copper for 30 min exhibited incomplete regeneration (Figure 2B,C), as did larvae treated with either concentration for 2 h (data not shown). These results are consistent with previous studies describing the effects of copper on hair cell regeneration [17] and damage to hair cell progenitors [40] in younger, 3 dpf embryos.

**Figure 3:**
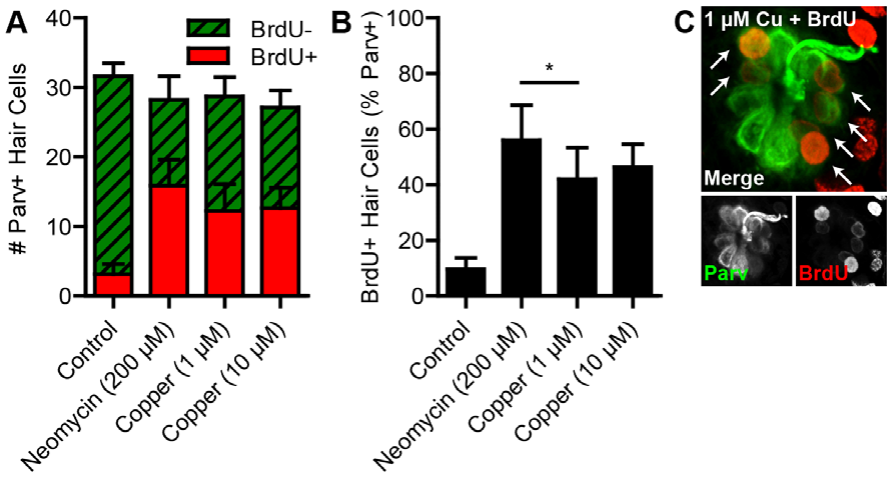
Hair cells are derived from proliferating progenitors. (A) Wild type larvae were treated at 5 dpf with neomycin or copper for 1 h followed by recovery in 5 mM BrdU until 24 hpt followed by fresh embryo medium. Greater numbers of BrdU-positive hair cells were observed in all ototoxin-treated groups at 72 hpt. (B) One-way ANOVA followed by a Tukey post-hoc analysis of the proportion of BrdU-positive hair cells revealed significant increases in all ototoxin-treated groups compared to control (*p*<0.001). No significant difference was observed between 1 and 10 μM copper that would suggest a dose-dependent effect on proliferative regeneration. A small but significant difference was observed between neomycin and 1 μM copper (*, *p*<0.05). *N* = 8 fish per group. Error bars are + SD. (C) Example neuromast at 72 hpt from a fish treated with 1 μM copper. Arrows mark BrdU-positive hair cells.

To examine whether treatment with neomycin or copper might bias regeneration toward proliferative or non-proliferative mechanisms, we used a pulse-chase paradigm to label new hair cells arising after damage with BrdU, a thymine analog that incorporates into DNA during S phase (Figure 3). Larvae were treated with 200 μM neomycin, 1 μM copper, or 10 μM copper for 1 h and then incubated in 5 mM BrdU for the first 23 h of recovery (24 h from the beginning of treatment). The BrdU was replaced with fresh embryo medium, and larvae recovered for an additional 48 h at which time they were fixed and stained with antibodies recognizing BrdU and parvalbumin to reveal mature hair cells derived from dividing precursors. Hair cells without BrdU staining were presumably derived from non-dividing progenitor cells or from progenitors that proliferated after exposure to BrdU.

All experimental conditions resulted in significantly more proliferation compared to controls (Figure 3A). One-way ANOVA revealed a highly significant main effect of treatment (*p*<0.0001). Approximately 40–50% of all hair cells in ototoxin-treated groups were BrdU-positive compared to ∼10% in control larvae, evidence of significant proliferative regeneration during the first 23 h of recovery (*p*<0.001; Figure 3B). Although there was a small but significant difference in the proportion of BrdU-positive hair cells between larvae treated with neomycin and 1 μM copper (*p*<0.05), there was no such difference between 1 μM and 10 μM copper. Taken together, these data support the idea that a large number of hair cells are derived from dividing precursors after damage from multiple ototoxins.

### Little Direct Transdifferentiation Occurs after Treatment with Neomycin or Copper

**Figure 4:**
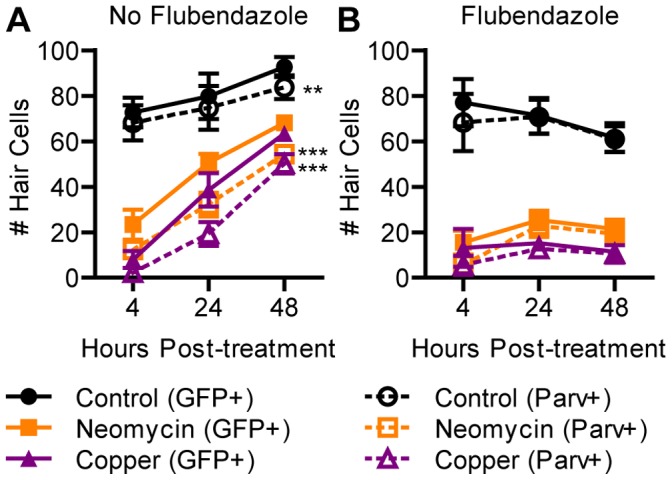
Hair cell regeneration is blocked by inhibition of mitosis. ET4:GFP larvae were treated at 5 dpf with neomycin or copper for 1 h and incubated in flubendazole for 48 h. (A) Those not treated with flubendazole continued to add mature hair cells. Two-way ANOVA found significantly more cells expressed GFP than parvalbumin (*p*<0.001), indicating the presence of hair cell precursors. Bonferroni post-hoc analysis confirmed a significant difference in control and ototoxin-treated groups between GFP- and parvalbumin-positive hair cell counts at 48 hpt (**, *p*<0.01; ***, *p*<0.001). (B) Those treated with flubendazole exhibited little or no increase in mature hair cells. There were at most 1 or 2 parvalbumin-negative precursors present per neuromast, indicating that undifferentiated precursors did not accumulate. *N* = 8 fish per group. Error bars are +/− SD.

Given that proliferative regeneration occurred after treatment with neomycin and with both low and high concentrations of copper, we sought to determine if preventing proliferation would uncover evidence of direct transdifferentiation. We treated 5 dpf larvae with neomycin or copper for 1 h and then incubated them in 5 μM flubendazole throughout the recovery period. Flubendazole blocks tubulin polymerization, preventing assembly of a mitotic spindle required for chromosome segregation [47], and has been identified as a drug that blocks hair cell regeneration [49]. Importantly, flubendazole shows little toxicity to hair cells themselves, removing a potential confounding effect of other mitotic inhibitors such as genistein and colchicine [42]. Larvae were collected up to 48 hours post-treatment (hpt) to establish a time course of regeneration with and without flubendazole exposure. All ototoxic treatments resulted in near-complete loss of hair cells in 5 dpf larvae and substantial recovery by 48 hpt (Figure 4A). Treatment with flubendazole alone had little effect on hair cell number in control fish but significantly impaired regeneration (Figure 4B; *p*<0.001). These results support the idea that hair cells are derived from dividing precursors and do not support the idea that latent transdifferentiation can compensate in the absence of proliferation.

**Figure 5:**
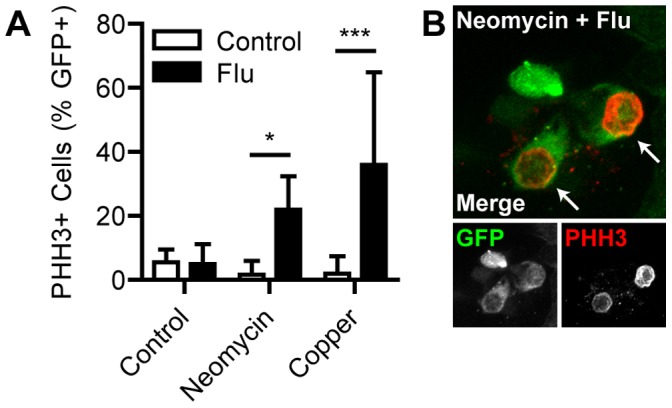
Flubendazole impairs division of hair cell progenitors. ET4:GFP larvae were treated at 5 dpf with neomycin or copper for 1 h and incubated in flubendazole for 24 h. Immunohistochemistry was performed for GFP and PHH3, which is upregulated during mitosis. (A) Two-way ANOVA followed by Bonferroni post-hoc analysis found significant increases in the proportion of PHH3-positive cells in neomycin- and copper-treated larvae when treated with flubendazole (*, *p*<0.05; ***, *p*<0.001), demonstrating that cell division was arrested in GFP-positive hair cell precursors. *N* = 8 fish per group. Error bars are + SD. (B) Example neuromast at 24 hpt from a fish treated with neomycin and flubendazole. Arrows mark PHH3-positive nuclei.

Impairment of microtubule assembly with flubendazole might impair differentiation of hair cells instead of blocking progenitor cell division. We therefore assayed the extent of regeneration during pharmacological inhibition of proliferation using transgenic ET4:GFP fish that express GFP in mature hair cells as well as pre-mitotic progenitors [37]. In the absence of flubendazole, the increase in GFP-positive cells significantly preceded the increase in parvalbumin-positive cells for control and both ototoxin-treated groups (Figure 4A; *p*<0.001). This is consistent with the acquisition of a progenitor cell fate before subsequent hair cell differentiation.However, there was no significant difference between GFP- and parvalbumin-positive cells in larvae treated with flubendazole (Figure 4B), indicating pre-mitotic progenitors did not accumulate. Analysis of larvae with antibodies against phosphohistone H3 (PHH3), which is expressed during mitosis, revealed incubation in flubendazole produced a significant increase in the proportion of PHH3-positive nuclei in GFP-positive cells during regeneration after neomycin (*p*<0.05; Figure 5) and copper (*p*<0.001). This result strongly suggests that flubendazole arrests hair cell progenitors during mitosis by interfering with spindle microtubules, not at a later stage of differentiation.

**Figure 6:**
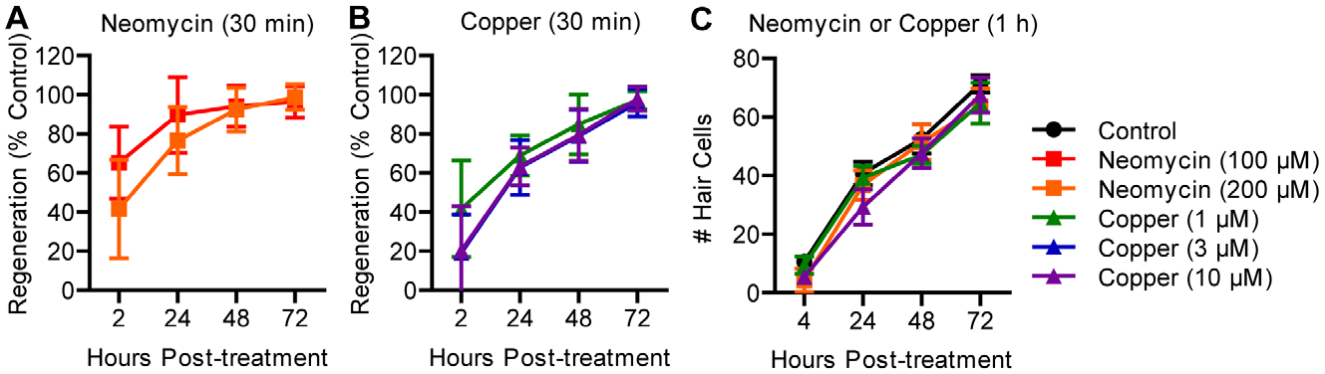
Regeneration is obscured by growth of the immature lateral line. (A, B) Wild type embryos were treated at 3 dpf with serial dilutions of neomycin or copper for 30 min. Hair cell regeneration was scored using FM 1-43FX and normalized to controls. Complete regeneration was observed in all groups. *N* = 14 fish per group. (C) Individual counts of hair cells labeled with antibodies against parvalbumin confirmed complete regeneration after 1 h treatment with copper or neomycin. However, all ototoxin-treated groups closely matched control embryos, which had few hair cells at the time of treatment. Instead of regeneration, most hair cell addition appeared related to early development of the lateral line. *N* = 8 fish per group. Error bars are +/− SD.

A previous report demonstrated non-proliferative addition of hair cells after copper treatment in younger, 3 dpf animals during late embryogenesis [38]. We therefore directly compared regeneration after different treatment conditions using neomycin or copper at 3 dpf. We observed that hair cell regeneration in all experimental groups was rapid and complete when compared to controls (Figure 6A,B). However, we also observed that control embryos added substantial numbers of hair cells over the course of the experiment (Figure 6C). The overall increase in hair cell number in ototoxin-treated groups closely followed the addition of hair cells in controls. This finding suggests that damage caused during embryonic growth can be compensated for by developmental mechanisms and many of the hair cells that arose after damage would have been added irrespective of toxin exposure.

### Proliferation is Required for Regeneration after Treatment with Gentamicin or Cisplatin

We next tested whether proliferative regeneration is the predominant mechanism of hair cell renewal after damage by other ototoxic compounds. Gentamicin can induce both rapid and delayed patterns of cell death, which appear to be regulated by intracellular pathways that may be distinct from those that follow neomycin treatment [33]. Cisplatin was shown to prevent regeneration in the chick inner ear [41]and has been used to study death of lateral line hair cells [32], [34], [43], but we were unaware of studies examining subsequent regeneration in zebrafish.

**Figure 7:**
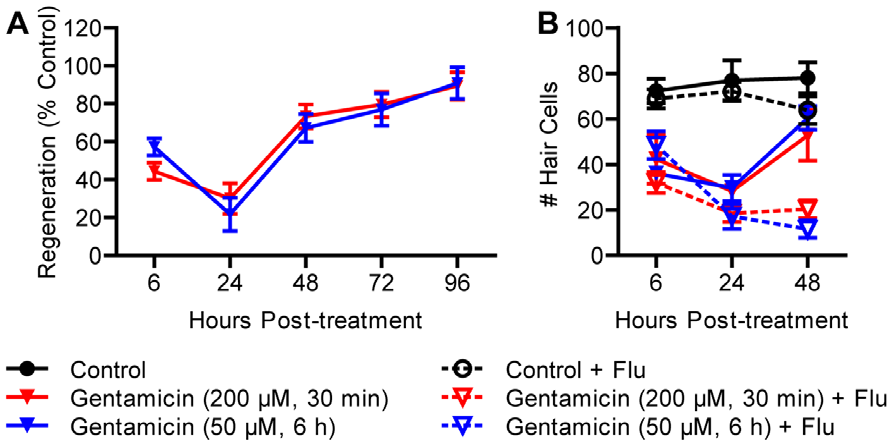
Proliferation is required for regeneration after treatment with gentamycin. (A) Wild type larvae were treated at 5 dpf with 200 μM gentamicin for 30 min (acute) or 50 μM gentamycin for 6 h (chronic) and allowed to recover for 96 h from the beginning of treatment. Gentamicin-induced hair cell death was delayed compared to neomycin. There was no dose-dependent effect on regeneration, which was complete by 96 hpt. (B) When fish were incubated in flubendazole during recovery, minimal regeneration was observed, suggesting that proliferation was required. *N* = 8 fish per group. Error bars are +/− SD.

To determine whether gentamicin treatment altered the rate of lateral line hair cell regeneration, we treated 5 dpf larvae with acute (200 μM, 30 min) or chronic (50 μM, 6 h) gentamicin and measured regeneration until 96 hpt. Approximately half of the hair cells remained at 6 h after the beginning of treatment and continued to decrease until 24 hpt (Figure 7A), consistent with previous results [33]. Complete regeneration was observed in both groups 72 h after this nadir. To determine if these two regimens differentially favored proliferation or direct transdifferentiation, we also incubated larvae in 5 μM flubendazole immediately after acute or chronic gentamicin treatments. Flubendazole significantly blocked regeneration in both groups when measured at 48 hpt (*p*<0.001; Figure 7B).

**Figure 8:**
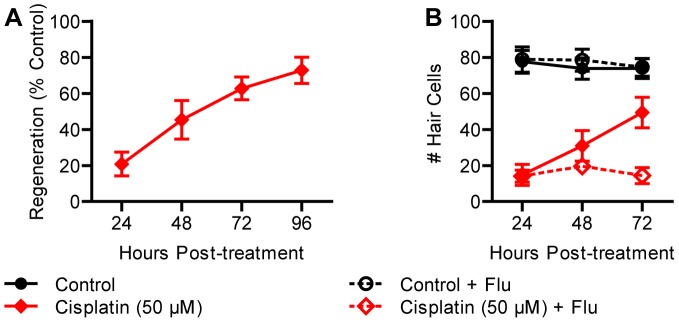
Proliferation is required for regeneration after treatment with cisplatin. (A) Wild type larvae were treated at 5 dpf with 50 μM cisplatin for 24 h and allowed to recover for 72 h (96 h from the beginning of treatment). Regeneration was considerable yet remained incomplete. (B) When fish were incubated in flubendazole during recovery, minimal regeneration was observed, suggesting that proliferation was required. *N* = 8 fish per group. Error bars are +/− SD.

We also determined the time course and extent of regeneration after cisplatin exposure. We treated 5 dpf larvae with chronic cisplatin (50 μM, 24 h) and measured regeneration until 96 hpt. Initial loss of hair cells was comparable to neomycin- and gentamicin-treated larvae, but regeneration was significantly delayed and remained incomplete at 96 hpt *(p*<0.001; Figure 8A). Incubating larvae in flubendazole during recovery significantly blocked the addition of new hair cells (*p*<0.001; Figure 8B). Thus, similar to copper and neomycin treatments, cisplatin and gentamicin treatments resulted in hair cell regeneration by a proliferative mechanism.

## Discussion

Our results support a model in which new lateral line hair cells are derived from dividing progenitors irrespective of the type of ototoxic damage initiating regeneration. By labeling proliferating cells with BrdU, we found extensive incorporation of this marker after a variety of treatment conditions. This is consistent with other studies that used time-lapse microscopy or BrdU incorporation to observe hair cell regeneration [15], [39], [42], [49]. Previous reports have found that nearly all BrdU-positive hair cells regenerate in pairs [39], suggesting that regeneration involves symmetric division of a progenitor into two daughter hair cells. Some progenitor cells may divide to form immature hair cell precursors prior to ototoxic damage, but incubation in BrdU both before and after neomycin treatment results in near complete incorporation of the marker [42]. Finally, live imaging studies of ET4:GFP larvae have followed the movement and division of GFP-positive cells from their earliest expression as pre-mitotic progenitors through division until their final differentiation as mature hair cells [37], [42]. Proliferation is not only sufficient but also necessary for hair cell regeneration.

We find that the drug flubendazole, an inhibitor of microtubule assembly [46], successfully blocked the production of new hair cells in regenerating neuromasts, consistent with reports using this and other mitotic inhibitors [42], [49]. An advantage to using flubendazole is that its application alone does not kill hair cells, which has complicated analysis of regeneration using other mitotic inhibitors [49]. An alternative is that it may block differentiation of precursor cells into hair cells. We hypothesize that one consequence would have resulted in an accumulation in ET4:GFP+ precursors as they failed to differentiate; this did not occur and instead an increase in ET4:GFP+ cells arrested in mitosis was observed. However we note that we cannot rule out that flubendazole might prevent the initiation of transdifferentiation through an unknown mechanism in addition to preventing proliferation. It also remains possible that if hair cell precursors were arrested at a stage earlier than M-phase, before initiation of DNA replication, they might be capable of transdifferentiation. However it has been previously reported that aphidicolin, which blocks initiation of DNA replication, also prevents hair cell regeneration [42].

Several other systems have been used to model auditory hair cell regeneration, and many of these make use of proliferative as well as non-proliferative regeneration. Although proliferative regeneration has been observed in birds [9], [10] and appears to be the dominant mechanism in the salamander [50], there is abundant evidence that early phases of direct transdifferentiation also occur in birds [20], [25], [51] and frogs [21], [24] and is a primary mechanism in newts [26]. There is also evidence of non-proliferative regeneration in the zebrafish utricular macula after laser ablation [52], suggesting a distinct response from the drug-induced hair cell loss described in the present study. These multiple mechanisms of regeneration mark a key difference between the zebrafish lateral line and other regenerating systems.

A few possible explanations exist for the absence of direct transdifferentiation during regeneration of lateral line hair cells. Rapid proliferation may be sufficient such that an alternate mechanism is unnecessary. Complete regeneration in the zebrafish lateral line occurs within days compared to one or two months before functional recovery in the chick auditory system [53], [54]. The early phase of direct transdifferentiation in the chick basilar papilla may serve to restore minimal auditory perception as rapidly as possible, relying on subsequent but slow proliferation for full regeneration. Lateral line hair cells are also thought to undergo continuous turnover [31], so it may be that proliferative mechanisms already in place to accomplish turnover are co-opted in response to acute damage. A similar mechanism was observed in the chick utricle, whereby an increase in hair cell death induced additional cell proliferation beyond that required for normal cell turnover [55].

We found that in addition to blocking hair cell production, flubendazole prevented the accumulation of additional ET4:GFP-positive progenitors. This suggests that a mechanism responsible for producing new progenitors is sensitive to the rate of hair cell differentiation. Notch signaling has been implicated in regulating interactions among progenitors and differentiating hair cells. A recent report found that progenitors acquire their cell fate without dividing and in regions of low Notch expression located in polar compartments on opposite ends of the neuromast [42]. We previously demonstrated that Notch signaling regulates the extent of differentiation of regenerating hair cells; as cells differentiate they prevent their neighbors from doing so, as they do during development [39], [56], [57]. If flubendazole interfered with transdifferentiation, we might expect progenitors to continue forming in the absence of lateral inhibition. There may also be other signals produced by post-mitotic hair cell precursors that influence this rate-limiting step. Flubendazole could interfere with the production of additional progenitors from dividing support cells elsewhere in the neuromast, but this would probably not affect regeneration until after the existing population of support cells was exhausted.

A major question is what mechanisms would need to be restored in mammals to promote hair cell regeneration. Although direct transdifferentiation seems simpler, it is not necessarily clear if proliferation or transdifferentiation provides a better opportunity to replace the large numbers of sensory hair cells required to restore auditory function in humans. Support cells undergo terminal mitosis in the mammalian cochlea while those in the vestibular system continue to proliferate in limited amounts [3], [4]. We observed that hair cell regeneration in the lateral line was greatly delayed after treatment with cisplatin or copper, consistent with their secondary effects on support cells [40], [41]. This delay indicates that support cell survival is a rate-limiting factor in hair cell regeneration. It also provides evidence of a robust DNA repair mechanism in the lateral line able to overcome the post-treatment apoptosis of dividing cells observed in the chick inner ear [41].

With or without proliferation, differentiation of progenitors into sensory hair cells requires expression of the transcription factor *atoh1*, which specifies hair cell fate during both development and regeneration [58], [59] and is regulated through lateral inhibition established by Notch signaling [59]. Notch signaling is required for specification of prosensory domains during early development of the inner ear [60]–[62], and *atoh1* is upregulated prior to the division of progenitor cells during regeneration of the chick basilar papilla [63]. However, *atoh1* also can serve as a hindrance during hair cell regeneration. For example, *atoh1* is expressed in some support cells in the mature mouse utricle after damage, but these rarely become new hair cells. Inhibition of Notch signaling increases both the amount of *atoh1* expression and the number of cells that successfully transdifferentiate [64]. In some ways, regeneration of the lateral line recapitulates early development. The role of proliferation, like Notch-regulated expression of *atoh1*, is conserved during both phenomena. Therefore, one of the challenges to regenerating human auditory hair cells appears to be re-initiating these early developmental steps without disrupting the fully formed and complex structure of the inner ear.
